# Supplementary material for: TBK1-associated adapters TANK and AZI2 protect mice against TNF-induced cell death and severe autoinflammatory diseases
Source: Nat Commun. 2024 Nov 19;15:10013. doi: 10.1038/s41467-024-54399-4 (PMC11576971; doi:10.1038/s41467-024-54399-4)
Supplement: Supplementary file 1 — Supplementary information [file 41467_2024_54399_MOESM1_ESM.pdf]

TBK1-associated adaptors TANK and AZI2 protect mice against TNF-induced cell death and severe autoinflammatory diseases

Andrea Ujevic[1], Daniela Knizkova[1,2], Alzbeta Synackova[1], Michaela Pribikova[1], Tijana Trivic[1,3], Anna Dalinskaya[1], Ales Drobek[2], Veronika Niederlova[2], Darina Paprckova[2,3], Roldan De Guia[4], Petr Kasparek[4], Jan Prochazka[4], Juraj Labaj[4], Olha Fedosieieva[4], Bernhard Florian Roeck[5], Ondrej Mihola[6], Zdenek Trachtulec[6], Radislav Sedlacek[4], Ondrej Stepanek[2], Peter Draber[1,2,3*]

[1] Laboratory of Immunity & Cell Communication, Division BIOCEV, First Faculty of Medicine, Charles University, Vestec, Czech Republic

[2] Laboratory of Adaptive Immunity, Institute of Molecular Genetics of the Czech Academy of Sciences, Prague, Czech Republic

[3] Department of Immunobiology, University of Lausanne, Epalinges, Switzerland

[4] Czech Centre for Phenogenomics and Laboratory of Transgenic Models of Diseases, Institute of Molecular Genetics of the Czech Academy of Sciences, Vestec, Czech Republic

[5] Institute for Genetics, CECAD Cluster of Excellence, University of Cologne, Cologne, Germany

[6] Laboratory of Germ Cell Development, Division BIOCEV, Institute of Molecular Genetics of the Czech Academy of Sciences, Czech Republic

* Correspondence: peter.draber@unil.ch

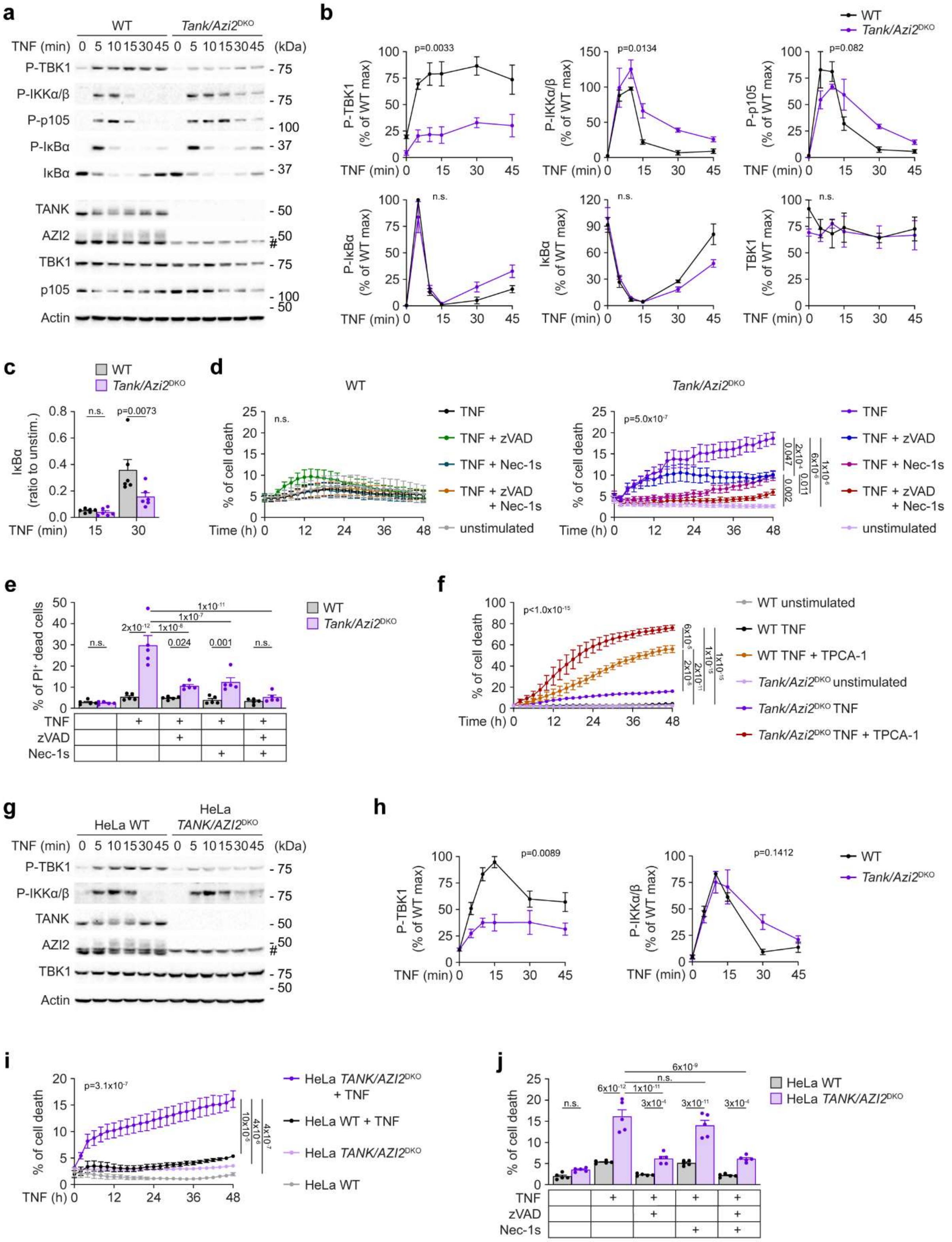

Supplementary Fig. 1. TANK and AZI2 regulate TNF-induced signaling and cell death.
(a-b) Immunoblot analysis of lysates of ST2 WT or *Tank/Azi2*^{DKO} cells stimulated with TNF (100 ng/ml) as indicated. A representative experiment (a) and quantification of three independent experiments

normalized to the maximal response of WT cells, mean ± SEM (b). Statistical analysis was based on the area under the curve (AUC) for a particular cell line in each experiment, unpaired two-tailed t-test.
(c) Degradation of IκB upon TNF (100 ng/ml) stimulation of ST2 WT or *Tank/Azi2*^{DKO} cells for 15 or 30 minutes. The protein expression was normalized to unstimulated cells. Mean + SEM from six independent experiments (based on data from Fig. 1c-d and Supplementary Fig. 1a-b), two-way ANOVA with Sidak's post-tests.
(d) ST2 WT or *Tank/Azi2*^{DKO} cells were stimulated with TNF (100 ng/ml) alone or in the presence of zVAD (20 μM), Nec-1s (20 μM), or both inhibitors. Induction of cell death was monitored every 2 h via the Incucyte imaging system. Mean ± SEM from six separate experiments using two different *Tank/Azi2*^{DKO} ST2 clones (correspond to Fig. 1h). Statistical analysis was based on the AUC for different cell lines and treatments in each experiment, one-way ANOVA (p-value shown) with Tukey´s post-tests.
(e) Analysis of cell death of ST2 WT or *Tank/Azi2*^{DKO} cells upon 24 hours of stimulation with TNF (250 ng/ml) alone or in the presence of zVAD (20 μM), Nec-1s (20 μM) or both inhibitors. Dead cells were detected by propidium iodide (PI) staining using flow cytometry. Data from five independent experiments are shown as mean + SEM, two-way ANOVA with Tukey´s post-tests.
(f) ST2 WT or *Tank/Azi2*^{DKO} cells were stimulated with TNF (100 ng/ml) alone or in the presence of TPCA-1 (5 μM). The induction of cell death was monitored and analyzed as in d. Mean ± SEM from eight separate experiments, one-way ANOVA (p-value shown) with Tukey´s post-tests.
(g-h) Immunoblot analysis of lysates of HeLa WT or *TANK/AZI2*^{DKO} cells stimulated with TNF (100 ng/ml) as indicated. A representative experiment (g) and quantification of four independent experiments, analyzed as in b, mean ± SEM, unpaired two-tailed t-test (h).
(i) HeLa WT and *TANK/AZI2*^{DKO} were unstimulated or stimulated with TNF (100 ng/ml). The induction of cell death was monitored and analyzed as in d. Mean ± SEM from five separate experiments, one-way ANOVA (p-value shown) with Tukey´s post-tests.
(j) HeLa WT and *TANK/AZI2*^{DKO} cells were stimulated with TNF (100 ng/ml) alone or in the presence of zVAD (20 μM), Nec-1s (20 μM), or both inhibitors. The induction of cell death after 48 h was measured using the Incucyte imaging system. Mean + SEM from five independent experiments, two-way ANOVA with Tukey´s post-tests.
n.s., not significant. #, nonspecific band.

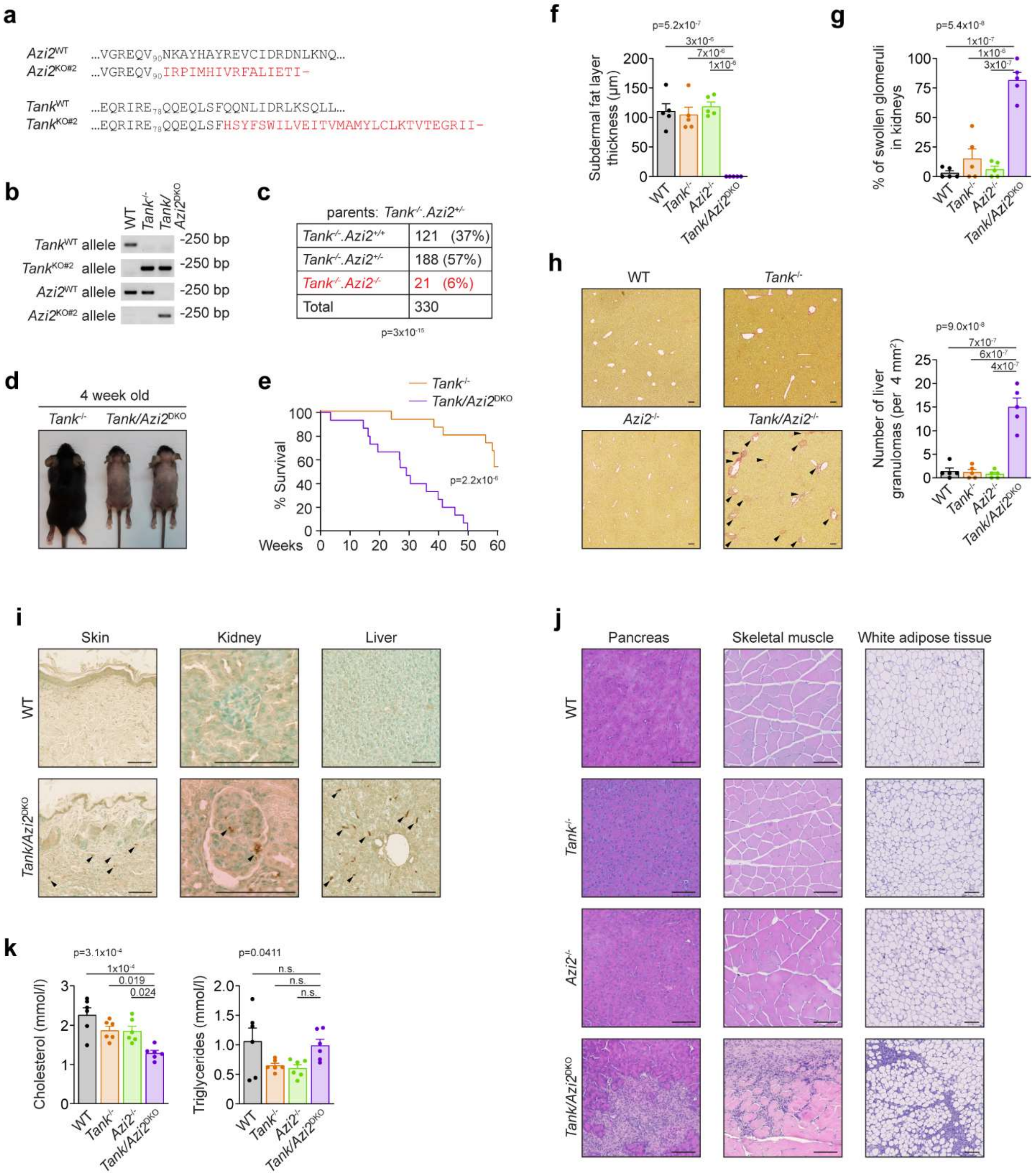

Supplementary Fig. 2. TANK and AZI2 protect against autoinflammatory disease in mice.

(a-b) Translations of DNA sequences (a) and an example of routine PCR genotyping (b) of the *Tank*$^{KO\#2}$ and *Azi2*$^{KO\#2}$ alleles from the second set of founder mice.

(c) Genotype and counts of pups born to parents harboring *Tank*$^{KO\#2}$ and *Azi2*$^{KO\#2}$ alleles as indicated, Chi-square test.

(d) Representative photographs of 4-week-old mice of the indicated genotype.

(e) Survival curves of mice of the indicated genotype from the second set of founder mice (n = 15 per group), log-rank Mantel-Cox test.

(f-h) Analysis of histological images (based on Fig. 2g-i) from mice of the indicated genotype (n = 5 per group). The average thickness of the subdermal fat layer (f), the proportion of swollen glomeruli in the kidney (g), and representative images of the liver with the indicated number of granulomas detected per 4 mm^2 sections (h). Mean + SEM, one-way ANOVA (p-value is displayed) with Tukey's post-tests.
(i) TUNEL staining of skin, kidney, and liver tissue sections isolated from 20-24-week-old WT or *Tank/Azi2*^{DKO} mice. TUNEL-positive cells are labeled by HRP-mediated oxidation of DAB (dark brown), and slides are counterstained with methyl green to detect nuclei. Representative images are based on analyzing samples from three mice per group. Arrows mark TUNEL-positive cells.
(j) H&E staining of tissue sections from the pancreas, skeletal muscle, and white adipose tissue isolated from 20-24-week-old mice of the indicated genotype. Representative images are based on analyzing samples from five mice per group.
(k) The concentration of cholesterol and triglycerides in the blood plasma isolated from 20-24-week-old mice of the indicated genotype (n = 6 per group). Mean + SEM, one-way ANOVA (p-value is displayed) with Tukey's post-tests.
n.s., not significant. The scale bar is 100 μm.

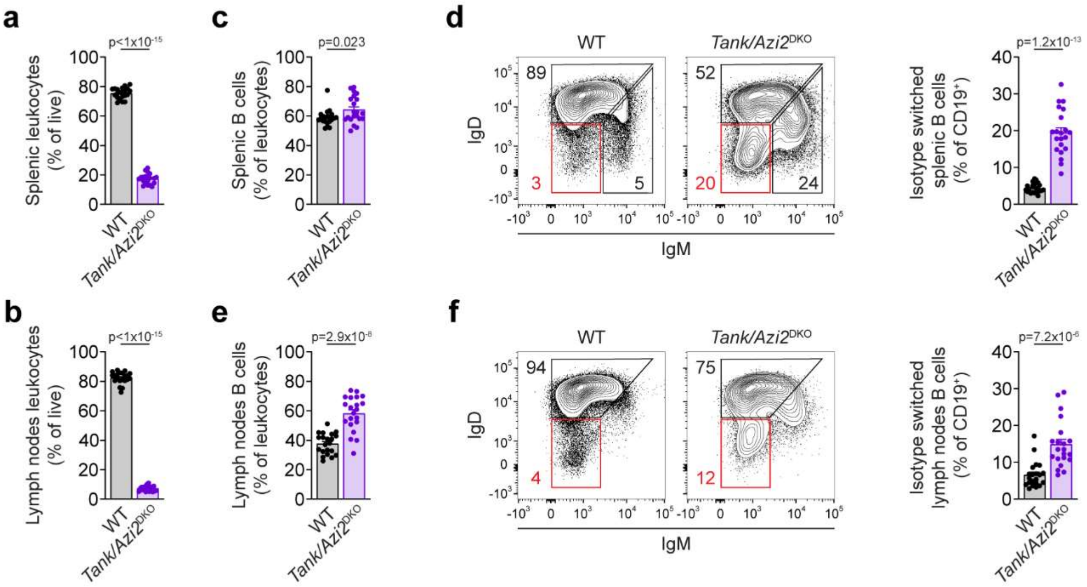

Supplementary Fig. 3. *Tank/Azi2*^{DKO} B cells have an intrinsic propensity to undergo isotype switching.
(a-f) Lethally irradiated Ly5.1 host mice were transplanted with bone marrow from Ly5.1/5.2 WT and Ly5.2 *Tank/Azi2*^{DKO} mice in a 1:1 ratio. Mice were sacrificed 12-14 weeks later, and cells from the spleen (a, c-d) and lymph nodes (b, e-f) were analyzed by flow cytometry. Percentage of leukocytes emanating from transplanted WT and *Tank/Azi2*^{DKO} bone marrow (a, b). The proportion of B cells ($CD19^+$) in the WT and *Tank/Azi2*^{DKO} leukocyte subset (c, e). Percentage of isotype-switched (IgM^-, IgD^-) WT and *Tank/Azi2*^{DKO} B cells (d, f). Each dot represents data from one animal based on three independent experiments (n = 21 per group). Mean + SEM, unpaired two-tailed t-test.

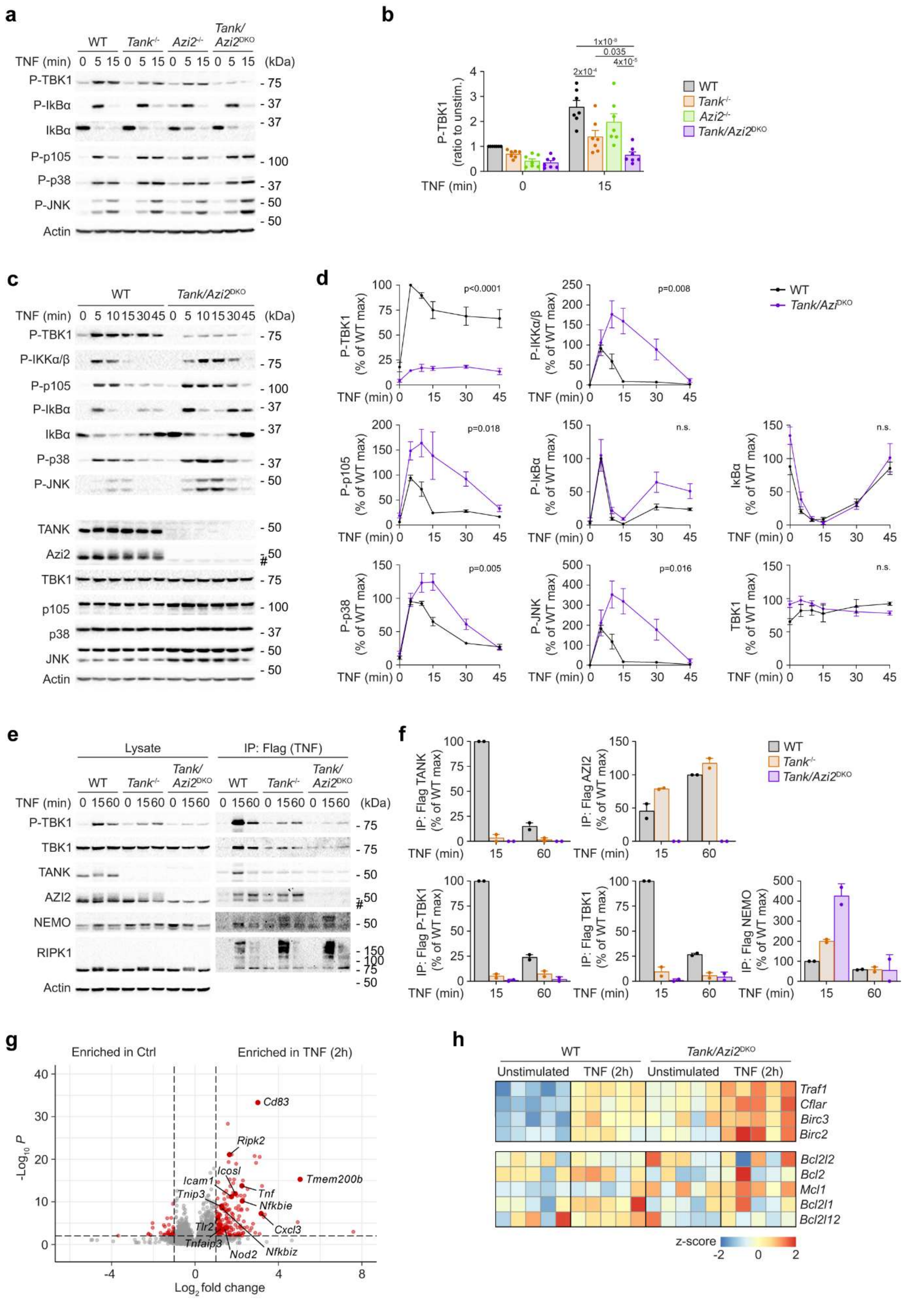
a
WT
Tank-/-
Azi2-/-
Tank/Azi2DKO
TNF (min) 0 5 15 0 5 15 0 5 15 0 5 15 (kDa)
P-TBK1 - 75
P-IkBα - 37
IkBα - 37
P-p105 - 100
P-p38 - 37
P-JNK - 50 - 50
Actin
b
P-TBK1 (ratio to unstim.)
TNF (min) 0 15
1x10-8
0.035
4x10-5
2x10-4
WT
Tank-/-
Azi2-/-
Tank/Azi2DKO
c
WT
Tank/Azi2DKO
TNF (min) 0 5 10 15 30 45 0 5 10 15 30 45 (kDa)
P-TBK1 - 75
P-IKKα/β - 75
P-p105 - 100
P-IkBα - 37
IkBα - 37
P-p38 - 37
P-JNK - 50
TANK - 50
Azi2 # 50
TBK1 - 75
p105 - 100
p38 - 37
JNK - 50 - 50
Actin
d
P-TBK1 (% of WT max) p<0.0001
P-IKKα/β (% of WT max) p=0.008
WT
Tank/AziDKO
P-p105 (% of WT max) p=0.018
P-IkBα (% of WT max) n.s.
IkBα (% of WT max) n.s.
P-p38 (% of WT max) p=0.005
P-JNK (% of WT max) p=0.016
TBK1 (% of WT max) n.s.
TNF (min) 0 15 30 45
e
Lysate
IP: Flag (TNF)
WT
Tank-/-
Tank/Azi2DKO
TNF (min) 0 15 60 0 15 60 0 15 60 0 15 60 0 15 60 0 15 60 (kDa)
P-TBK1 - 75
TBK1 - 75
TANK - 50
AZI2 # 50
NEMO - 50
RIPK1 - 150 - 100 - 75 - 50
Actin
f
IP: Flag TANK (% of WT max)
IP: Flag AZI2 (% of WT max)
WT
Tank-/-
Tank/Azi2DKO
IP: Flag P-TBK1 (% of WT max)
IP: Flag TBK1 (% of WT max)
IP: Flag NEMO (% of WT max)
TNF (min) 15 60
g
Enriched in Ctrl
Enriched in TNF (2h)
-Log10 P
Log2 fold change
Cd83
Ripk2
Tmem200b
Icosl
Icam1
Tnip3
Tnf
Nfkbie
Cxcl3
Tlr2
Tnfaip3
Nod2
Nfkbiz
h
WT
Tank/Azi2DKO
Unstimulated
TNF (2h)
Traf1
Cflar
Birc3
Birc2
Bcl2l2
Bcl2
Mcl1
Bcl2l1
Bcl2l12
z-score -2 0 2

Supplementary Fig. 4. TNF-induced TBK1 phosphorylation is impaired in *Tank/Azi2*^{DKO} cells.

(a-b) Immunoblot analysis of cell lysates from MEFs isolated from WT, *Tank*$^{-/-}$, *Azi2*$^{-/-}$, or *Tank/Azi2*^{DKO} mice. The cells were stimulated with TNF (100 ng/ml), as indicated. A representative experiment (a) and quantification of seven independent experiments normalized to unstimulated WT cells (b). Mean + SEM, two-way ANOVA with Tukey's post-tests.

(c-d) Immunoblot analysis of cell lysates from MEFs isolated from WT or *Tank/Azi2*^{DKO} mice. The cells were stimulated with TNF (100 ng/ml) as indicated. A representative experiment (c) and quantification of four independent experiments normalized to the maximal response of WT cells, mean ± SEM (d). Statistical analysis was based on the AUC for particular cell lines in each experiment, unpaired two-tailed t-test.

(e-f) Immunoblot analysis of TNF-RSC isolated from MEFs of the indicated genotype. Cells were stimulated with SF-TNF (500 ng/ml) for different time points, and lysates were subjected to anti-Flag immunoprecipitation. As a control, SF-TNF was added post-lysis to non-stimulated samples. A representative experiment (e) and quantification of recruitment of indicated proteins to TNF-RSC from two independent experiments, mean + SEM (f).

(g-h) RNAseq analysis of WT and *Tank/Azi2*^{DKO} BMDM stimulated for 2 h with TNF (100 ng/ml). The BMDMs were independently isolated from 3 pairs of animals, and RNA was isolated in five separate experiments. The volcano plot shows differentially expressed genes in TNF-stimulated versus unstimulated cells (log_2 fold change > 1 and padj < 0.01) (g). The heatmap shows the expression of selected genes (h).
n.s., not significant. #, nonspecific band.

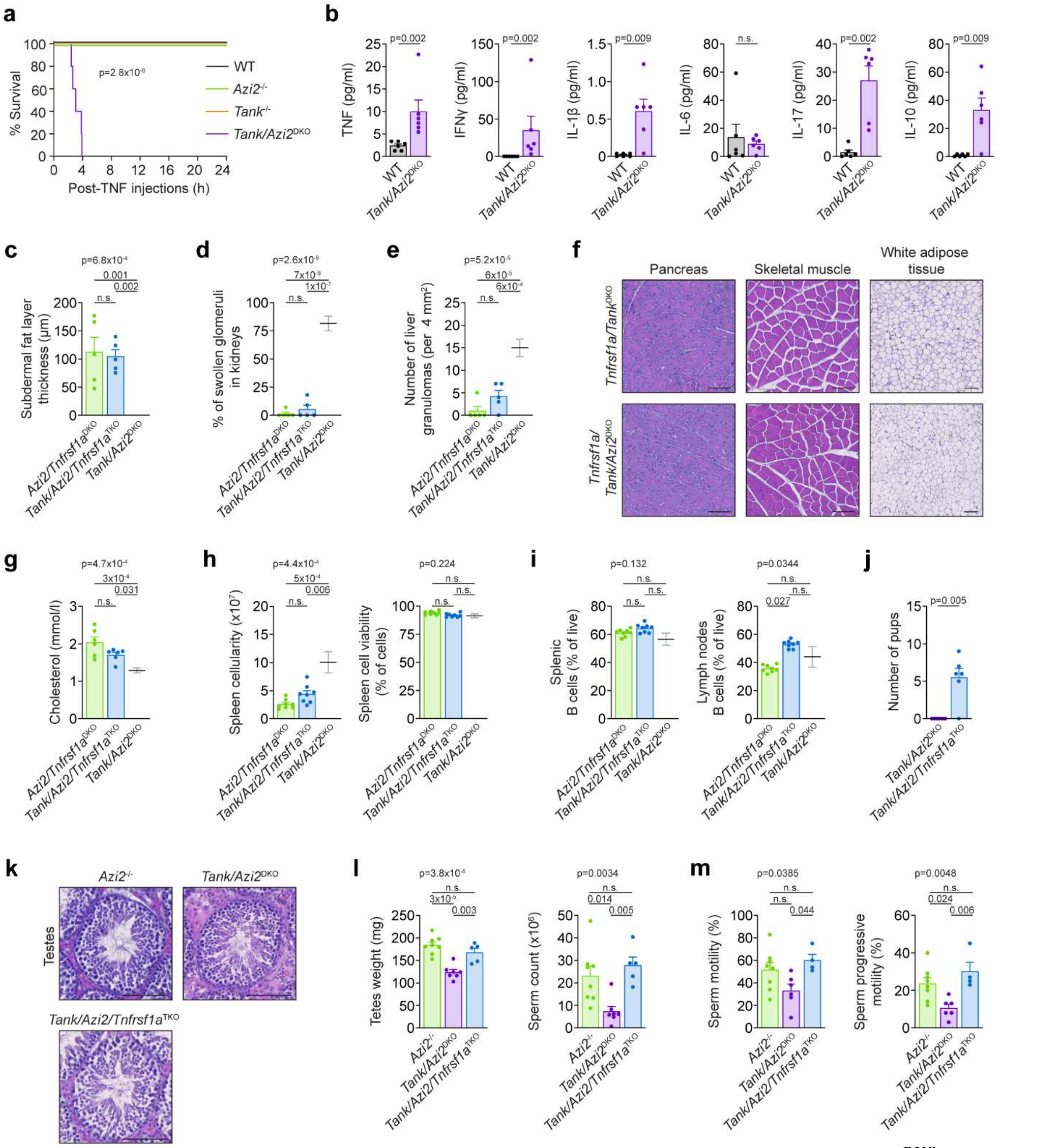

Supplementary Fig. 5. TNFR1 drives autoinflammation and male sterility in *Tank/Azi2*^{DKO} mice.

(a) Survival of 10-12-week-old mice of the indicated genotype upon intravenous injection of TNF (5 µg per mouse) (n = 6 for WT and 5 for the other genotypes), log-rank Mantel-Cox test.

(b) The concentration of the indicated cytokines in the blood plasma isolated from 20-24-week-old WT or *Tank/Azi2*^{DKO} mice (n = 6 per group), mean + SEM, two-tailed Mann-Whitney U test.

(c-e) Analysis of histological images (based on Fig. 5e-h) from mice of the indicated genotype (n = 5 per group). The average thickness of the subdermal fat layer (c), the proportion of swollen glomeruli in the kidney (d), and the number of liver granulomas detected per 4 mm^2 sections (e). Mean + SEM, one-way ANOVA (p-value shown), followed by Tukey's post-tests.

(f) H&E staining of tissue sections from the pancreas, skeletal muscle, and white adipose tissue isolated from 20-24-week-old mice of the indicated genotype. Representative images shown are based on analyzing samples from five mice per group.
(g) The cholesterol concentration in the blood plasma isolated from 20-24-week-old mice of the indicated genotype (n = 6 per group), mean + SEM, one-way ANOVA (p-value shown), followed by Tukey's post-tests.
(h) Flow cytometry analysis of cellularity and viability of the spleen isolated from 8-12-week-old mice of the indicated genotype (n = 8 per group), mean + SEM, one-way ANOVA (p-value shown), followed by Tukey's post-tests.
(i) Flow cytometry analysis of B cells ($CD19^+$) in the spleen and lymph nodes isolated from 8-12-week-old mice of the indicated genotype (n = 8 per group), mean + SEM, one-way ANOVA (p-value shown), followed by Tukey's post-tests.
(j) The number of pups born to 6-15-week-old males of the indicated genotype. Males (n = 6 per group) were caged with 6-15-week-old WT females until the first pups were born or for 40 days, at which point the males were separated, and females were kept for at least four additional weeks. The first litter size from each breeding pair is shown. 0 indicates no litter. Mean + SEM, two-tailed Mann-Whitney U test.
(k) H&E staining of testicular sections from 20-24-week-old mice of the indicated genotype. Representative images shown are based on analyzing samples from three mice per group.
(l-m) Analysis of the testes isolated from 11-23-week-old male mice of the indicated genotypes (n = 8 for *Azi2*$^{-/-}$, 7 for *Tank/Azi2*^{DKO}, 5 for *Tank/Azi2/Tnfrsf1a*^{TKO}). Weight of paired testicles and epididymal sperm count (l). The percentage of motility and progressive motility was analyzed via computer-assisted sperm analysis (m). Mean + SEM, one-way ANOVA (p-value shown), followed by Tukey's post-tests.
In gray is the comparison with *Tank/Azi2*^{DKO} mice (based on Fig. 3 and Supplementary Fig. 2). n.s., not significant. The scale bar is 100 µm.

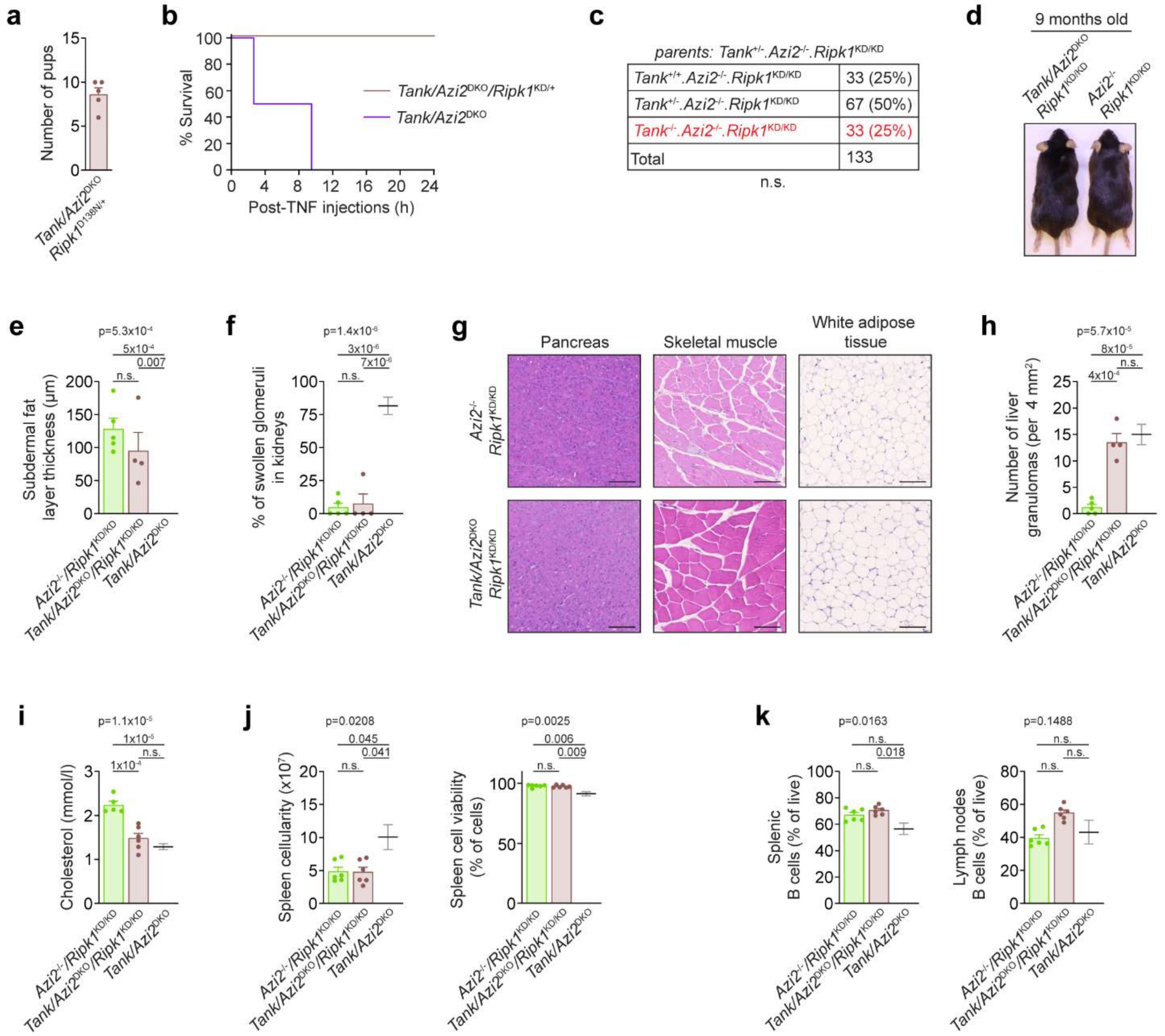

Supplementary Fig. 6. RIPK1 kinase activity promotes autoinflammation in *Tank/Azi2*^{DKO} mice.

(a) The number of pups born to 8-12-week-old *Tank/Azi2*^{DKO}*/Ripk1*$^{\mathrm{KD/+}}$ males. Males (n = 5 per group) were caged with WT females. Each breeding pair had litter within four weeks. The litter size is shown, mean + SEM.

(b) Survival of 8-12-week-old *Tank/Azi2*^{DKO}*/Ripk1*$^{\mathrm{KD/+}}$ (n = 5) or *Tank/Azi2*^{DKO} mice (n = 2) upon intravenous injection of TNF (5 µg per mouse).

(c) Genotype of pups born to *Tank*$^{+/-}$*.Azi2*$^{-/-}$*.Ripk1*$^{\mathrm{KD/KD}}$ parents. **(d)** Representative photographs of 9-month-old mice of the indicated genotype, Chi-square test.

(e-f) Analysis of histological images (based on Fig. 6d-e) from mice of the indicated genotype (n = 5 for *Azi2*$^{-/-}$*.Ripk1*$^{\mathrm{KD/KD}}$, 4 for *Tank*/*Azi2*^{DKO}/*Ripk1*$^{\mathrm{KD/KD}}$). The average thickness of the subdermal fat layer (e) and proportion of swollen glomeruli in the kidney (f), mean + SEM, one-way ANOVA (p-value shown), followed by Tukey's post-tests.

(g) H&E staining of tissue sections from the pancreas, skeletal muscle, and white adipose tissue isolated from 20-24-week-old mice of the indicated genotype. Representative images are based on analyzing samples from five mice per group.

(h) Number of liver granulomas detected per 4 mm^2 histological sections (based on Fig. 6f) from mice of the indicated genotype (n = 5 for *Azi2*$^{-/-}$.*Ripk1*$^{KD/KD}$, 4 for *Tank*/*Azi2*^{DKO}/*Ripk1*$^{KD/KD}$), mean + SEM, one-way ANOVA (p-value shown), followed by Tukey's post-tests.
(i) The concentration of cholesterol in the blood plasma isolated from 20-24-week-old *Azi2*$^{-/-}$/*Ripk1*$^{KD/KD}$ mice (n = 5) or *Tank/Azi2*^{DKO}/*Ripk1*$^{KD/KD}$ mice (n = 6), mean + SEM, one-way ANOVA (p-value shown), followed by Tukey's post-tests.
(j) Flow cytometry analysis of cellularity and viability of the spleen isolated from 8-13-week-old mice of the indicated genotype (n = 8 only for *Tank/Azi2*^{DKO}, 6 for the other genotypes), mean + SEM, one-way ANOVA (p-value shown), followed by Tukey's post-tests.
(k) Flow cytometry analysis of B cells ($CD19^+$) in the spleen and lymph nodes isolated from 8-12-week-old mice of the indicated genotype (n = 6 per group), mean + SEM, one-way ANOVA (p-value shown), followed by Tukey's post-tests.
In gray is the comparison with *Tank/Azi2*^{DKO} mice (based on Fig. 3 and Supplementary Fig. 2). n.s., not significant. The scale bar is 100 µm.

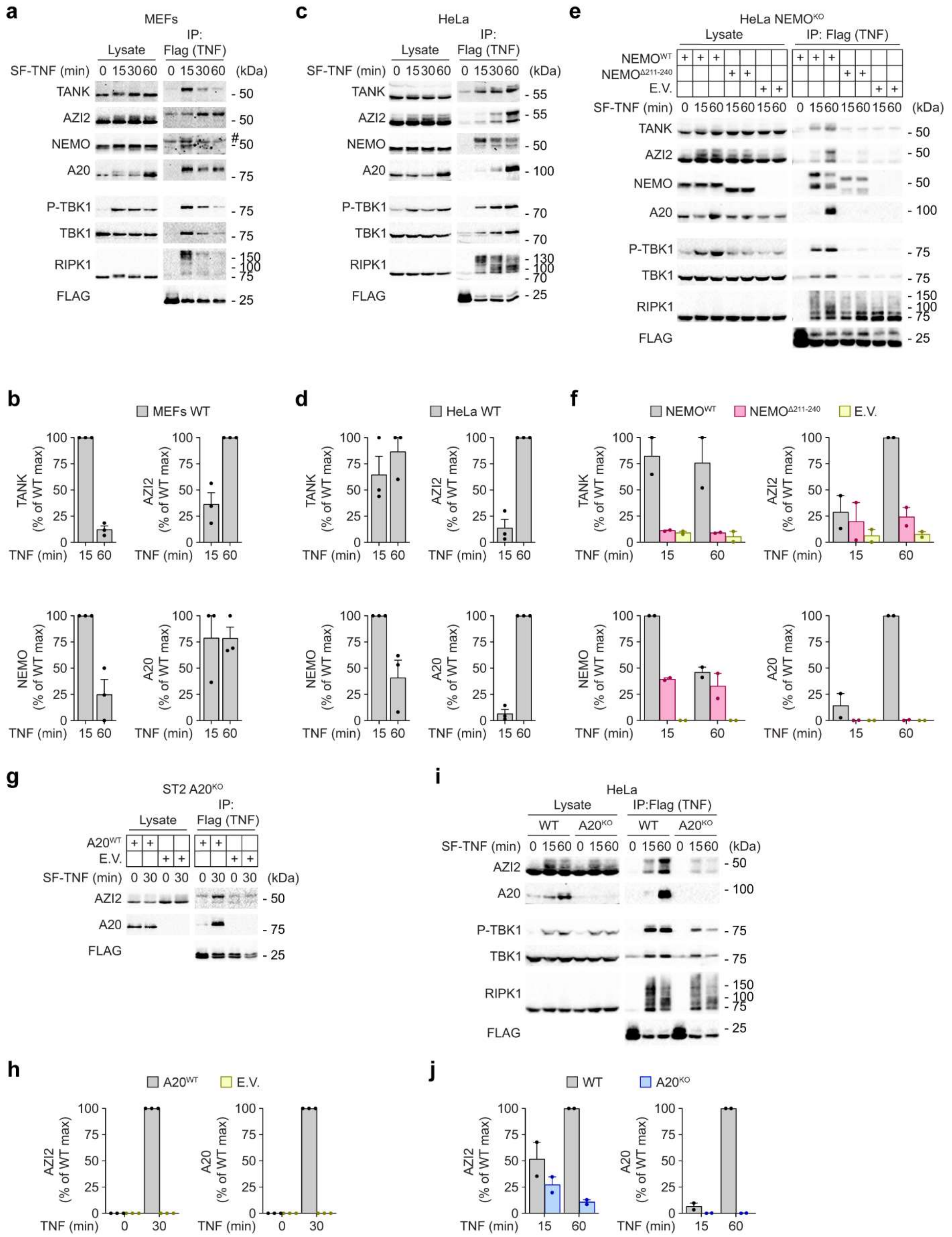

Supplementary Fig. 7. TANK and AZI2 differ in their mechanism of recruitment to TNF-RSC.
(a-j) Immunoblot analysis of TNF-RSC isolated from MEFs (a-b), HeLa (c-d), NEMO-deficient HeLa cells reconstituted with NEMO^{WT}, NEMO$^{\Delta211\text{-}240}$ or empty vector (E.V.) (e-f), A20-deficient ST2 cells

reconstituted with $A20^{WT}$ or E.V. (g-h), or HeLa WT or $A20^{KO}$ (i-j). Cells were stimulated with Strep-Flag-TNF (SF-TNF) (500 ng/ml) for different time points, and lysates were subjected to anti-Flag immunoprecipitation. As a control, SF-TNF was added post-lysis to non-stimulated samples. Representative experiments (a, c, e, g, i) and quantification of recruitment of indicated proteins to TNF-RSC from three (b, d, h) or two (f, j) independent experiments normalized to the maximal response of WT cells, mean + SEM.
#, nonspecific band.

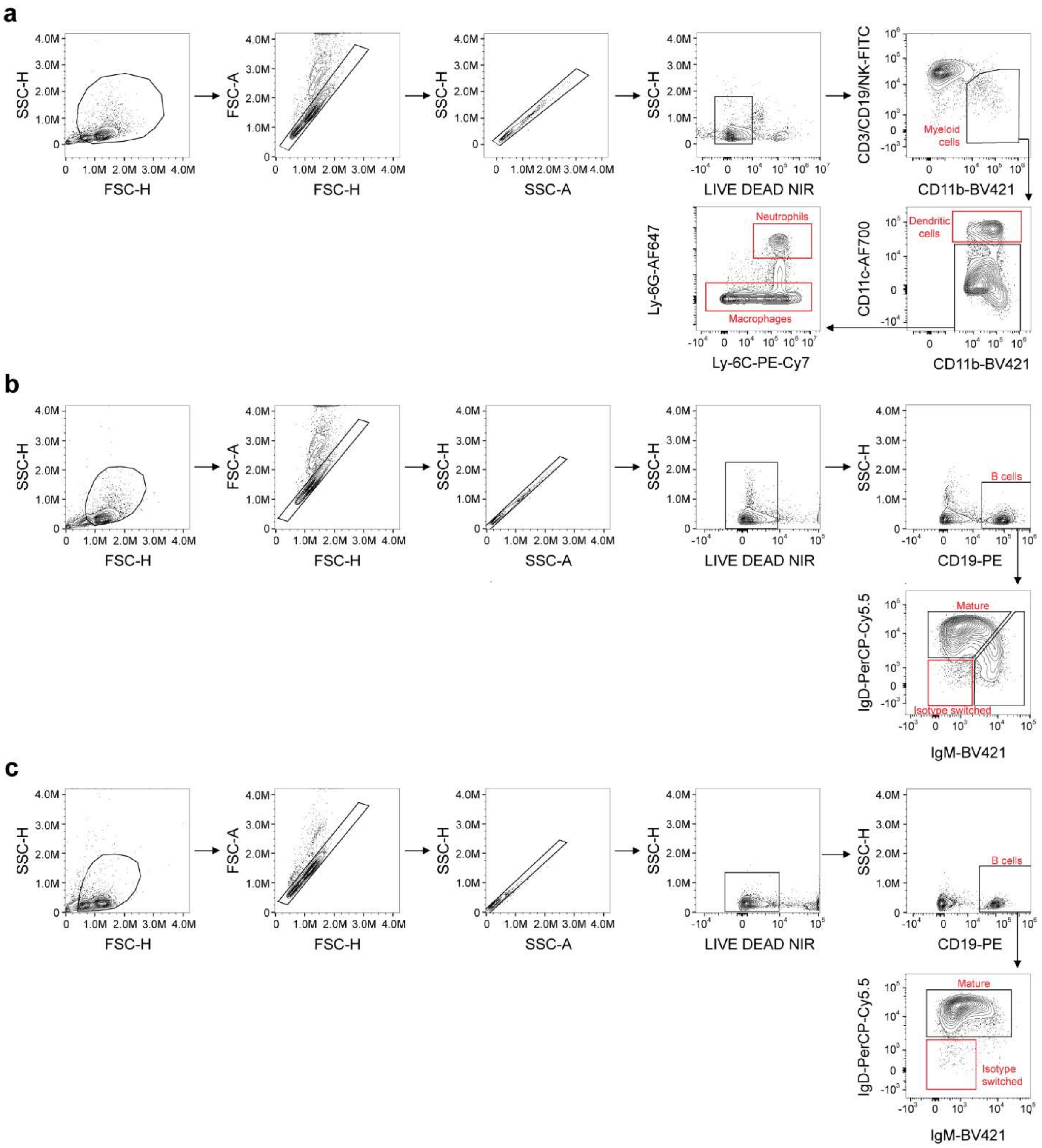

Supplementary Fig. 8. Gating strategy.

(a-c) Flow cytometry gating strategy for splenic myeloid cells employed in Fig. 3e (a), splenic B cells employed in Fig. 3g, 5n, 6m and Supplementary Fig. 3f-g (b), and lymph nodes B cells used in Fig. 3i, 5o, 6n and Supplementary Fig. 3h-i (c).
