# Supplementary material for: TBK1-associated adapters TANK and AZI2 protect mice against TNF-induced cell death and severe autoinflammatory diseases
Source: Nat Commun. 2024 Nov 19;15:10013. doi: 10.1038/s41467-024-54399-4 (PMC11576971; doi:10.1038/s41467-024-54399-4)
Supplement: Supplementary file 3 — Reporting Summary [file 41467_2024_54399_MOESM3_ESM.pdf]

nature portfolio

Corresponding author(s): Peter Draber

Last updated by author(s): Sep 28, 2024

# Reporting Summary

Nature Portfolio wishes to improve the reproducibility of the work that we publish. This form provides structure for consistency and transparency in reporting. For further information on Nature Portfolio policies, see our Editorial Policies and the Editorial Policy Checklist.

## Statistics

For all statistical analyses, confirm that the following items are present in the figure legend, table legend, main text, or Methods section.

| n/a | Confirmed | |
|---|---|---|
| ☐ | ☒ | The exact sample size ($n$) for each experimental group/condition, given as a discrete number and unit of measurement |
| ☐ | ☒ | A statement on whether measurements were taken from distinct samples or whether the same sample was measured repeatedly |
| ☐ | ☒ | The statistical test(s) used AND whether they are one- or two-sided<br>*Only common tests should be described solely by name; describe more complex techniques in the Methods section.* |
| ☒ | ☐ | A description of all covariates tested |
| ☐ | ☒ | A description of any assumptions or corrections, such as tests of normality and adjustment for multiple comparisons |
| ☐ | ☒ | A full description of the statistical parameters including central tendency (e.g. means) or other basic estimates (e.g. regression coefficient) AND variation (e.g. standard deviation) or associated estimates of uncertainty (e.g. confidence intervals) |
| ☐ | ☒ | For null hypothesis testing, the test statistic (e.g. $F$, $t$, $r$) with confidence intervals, effect sizes, degrees of freedom and $P$ value noted<br>*Give P values as exact values whenever suitable.* |
| ☒ | ☐ | For Bayesian analysis, information on the choice of priors and Markov chain Monte Carlo settings |
| ☒ | ☐ | For hierarchical and complex designs, identification of the appropriate level for tests and full reporting of outcomes |
| ☒ | ☐ | Estimates of effect sizes (e.g. Cohen's $d$, Pearson's $r$), indicating how they were calculated |

*Our web collection on statistics for biologists contains articles on many of the points above.*

## Software and code

Policy information about availability of computer code

| | |
|---|---|
| Data collection | No software for data collection was used. |
| Data analysis | Cytometry data were analyzed using FlowJo software v10.10.0 (BD Biosciences).<br>Histology sections were analyzed using ZEN v3.4 software (Zeiss).<br>Immunoblotting data were analyzed using Image Lab v6.1 (Bio-Rad).<br>Statistical analysis was performed and graphs were plotted using Prism v10.2.3 (GraphPad).<br>Induction of cell death measured on Incucyte was analyzed by custom made software. |

For manuscripts utilizing custom algorithms or software that are central to the research but not yet described in published literature, software must be made available to editors and reviewers. We strongly encourage code deposition in a community repository (e.g. GitHub). See the Nature Portfolio guidelines for submitting code & software for further information.

## Data

Policy information about availability of data

All manuscripts must include a data availability statement. This statement should provide the following information, where applicable:

- Accession codes, unique identifiers, or web links for publicly available datasets
- A description of any restrictions on data availability
- For clinical datasets or third party data, please ensure that the statement adheres to our policy

The bulk RNA sequencing data have been deposited in Sequence Read Archive (SRA) under the accession code PRJNA1095284 [https://www.ncbi.nlm.nih.gov/bioproject/PRJNA1095284]. The code for the custom-made software used to determine the percentage of cell death in high-throughput live cell imaging data can be accessed in the public repository Zenodo [https://zenodo.org/records/13774183]. Source data are provided with this paper.

## Research involving human participants, their data, or biological material

Policy information about studies with human participants or human data. See also policy information about sex, gender (identity/presentation), and sexual orientation and race, ethnicity and racism.

| | |
|---|---|
| Reporting on sex and gender | The manuscript does not contain research involving human participants. |
| Reporting on race, ethnicity, or other socially relevant groupings | The manuscript does not contain research involving human participants. |
| Population characteristics | The manuscript does not contain research involving human participants. |
| Recruitment | The manuscript does not contain research involving human participants. |
| Ethics oversight | The manuscript does not contain research involving human participants. |

Note that full information on the approval of the study protocol must also be provided in the manuscript.

# Field-specific reporting

Please select the one below that is the best fit for your research. If you are not sure, read the appropriate sections before making your selection.

☒ Life sciences ☐ Behavioural & social sciences ☐ Ecological, evolutionary & environmental sciences

For a reference copy of the document with all sections, see nature.com/documents/nr-reporting-summary-flat.pdf

# Life sciences study design

All studies must disclose on these points even when the disclosure is negative.

| | |
|---|---|
| Sample size | The sample size was determined based on our previous experience with similar type of experiments. |
| Data exclusions | No data were excluded from analyses. |
| Replication | The number of replications for each experiment is specified in each figure. All experiments were repeated several times, as specified in the manuscript. |
| Randomization | Mice were allocated to particular test groups based on their genotype. Experiments using cell lines were not randomized, the design of individual experiments allowed to control for covariants. |
| Blinding | The mice were analyzed in a blinded manner, with the exception of the Tank/Azi2DKO mice, which are distinguishable due to delay in hair growth and ongoing dermatitis.<br>In the case of experiments using cell lines, the blinding was not performed as different cell lines were compared side by side. |

# Reporting for specific materials, systems and methods

We require information from authors about some types of materials, experimental systems and methods used in many studies. Here, indicate whether each material, system or method listed is relevant to your study. If you are not sure if a list item applies to your research, read the appropriate section before selecting a response.

## Materials & experimental systems

| n/a | Involved in the study |
| --- | --- |
| ☐ | ☒ Antibodies |
| ☐ | ☒ Eukaryotic cell lines |
| ☒ | ☐ Palaeontology and archaeology |
| ☐ | ☒ Animals and other organisms |
| ☒ | ☐ Clinical data |
| ☒ | ☐ Dual use research of concern |
| ☒ | ☐ Plants |

## Methods

| n/a | Involved in the study |
| --- | --- |
| ☒ | ☐ ChIP-seq |
| ☐ | ☒ Flow cytometry |
| ☒ | ☐ MRI-based neuroimaging |

# Antibodies

Antibodies used

All antibodies used are listed in Supplementary Data Table 2.

Validation

Most antibodies employed in this study are commonly used by us and other laboratories. The validation of individual antibodies can be found on the manufacturer's website. TANK, AZI2, NEMO, and A20 antibodies were validated in knockout cell lines.

# Eukaryotic cell lines

Policy information about cell lines and Sex and Gender in Research

Cell line source(s)

ST2 cells were kindly provided by J. Balounova, and HeLa, HEK293FT, ØNX-Eco, and ØNX-Ampho cells were kindly provided by T. Brdicka (both from the Institute of Molecular Genetics, Prague, Czech Republic). MEFs were derived from E11.5 mouse embryos and immortalized by transduction with SV40 large T antigen.

Authentication

Listed cells are commonly used in our lab. No additional authentication was performed.

Mycoplasma contamination

All cell lines were regularly tested for the presence of Mycoplasma using the Mycoplasmacheck services (Eurofins Genomics).

Commonly misidentified lines
(See ICLAC register)

No commonly misidentified cell lines were used in the study.

# Animals and other research organisms

Policy information about studies involving animals; ARRIVE guidelines recommended for reporting animal research, and Sex and Gender in Research

Laboratory animals

The murine strains utilized in this study were generated at the Czech Centre for Phenogenomics at IMG. To introduce targeted indel mutations into the Tank and Azi2 genes, a mixture containing Cas9 mRNA (100 ng/ml) and target-specific sgRNA (50 ng/ml) was microinjected into zygotes derived from C57BL6/N mice. Zygote electroporation was employed to deliver sgRNAs targeting Tnfrsf1a and Ripk1 genes. In the case of producing the RIPK1KD allele harboring the inactivating D138N mutation, a single-stranded DNA template (10 µM) was included. The resulting embryos were then implanted into foster mothers. Genomic DNA was isolated from tail biopsies of newborn mice, and the DNA sequence surrounding the CRISPR/Cas9 recognition site was sequenced. The selected founders were backcrossed onto the C57BL/6J background for at least three generations.
The age of mice is specified for individual experiments. Both males and females were used.

Wild animals

This study did not involve wild animals.

Reporting on sex

Animals of both sexes were used.

Field-collected samples

This study did not involve field-collected samples.

Ethics oversight

All animal protocols for the mice experiments were approved by the Resort Professional Commission for Approval of Projects of Experiments on Animals of the Czech Academy of Sciences, Czech Republic, project number 36/2020.

Note that full information on the approval of the study protocol must also be provided in the manuscript.

## Flow Cytometry

### Plots

Confirm that:

☒ The axis labels state the marker and fluorochrome used (e.g. CD4-FITC).

☒ The axis scales are clearly visible. Include numbers along axes only for bottom left plot of group (a 'group' is an analysis of identical markers).

☒ All plots are contour plots with outliers or pseudocolor plots.

☒ A numerical value for number of cells or percentage (with statistics) is provided.

### Methodology

| | |
|---|---|
| Sample preparation | To analyze of immune cell populations, 8-12 week old mice were sacrificed, and their spleen and peripheral lymph nodes were removed and minced to obtain single-cell suspensions. In the case of the spleen, red blood cells were lysed in ACK buffer (150 mM NH4Cl, 10 mM KHCO3, 0.1 mM EDTA- Na2, pH 7.4). Cells were resuspended in FACS buffer and stained on ice with LIVE/DEAD near-IR dye (Life Technologies). To analyze B cells, the samples were stained with the following antibody panel: CD19-PE, IgM-BV421, IgD-PerCP_Cy5.5, CD23-APC, and CD1d-PE-Cy7. For the analysis of myeloid cells, the samples were stained with the following antibody panel: CD3-FITC, CD19-FITC, NK1.1-FITC, CD11b-BV421, CD11c-AF700, Ly6C-PE-Cy7, and Ly6G-AF647. All the fluorescently labeled antibodies are listed in Supplementary Data Table 2. Samples were measured on Cytek Aurora flow cytometer.<br><br>For the analysis of cell death using flow cytometry, WT or TANK/AZI2 DKO ST2 cells in DMEM with 10% FSC were pretreated with the caspase inhibitor zVAD (20µM) and/or RIPK1 inhibitor Necrostatin-1s (20µM) for 15 minutes at 37°C. Subsequently, mouse TNF (250 ng/ml) was added as indicated. Cells were incubated for 24 hours. The induction of dead cells was determined by detecting the percentage of propidium-positive cells using the BriCyte E3 flow cytometer (Mindray). |
| Instrument | Aurora (Cytek) or BriCyte E3 (Mindray) flow cytometers. |
| Software | Data were analyzed using FlowJo software (TreeStar) |
| Cell population abundance | Retrovirally transduced cells were sorted as GFP positive, and the purity was regularly tested via FACS. |
| Gating strategy | B cells (CD19+) were gated to identify isotype-switched cells (IgM-, IgD-). Myeloid cells (CD3-, CD19-, NK1.1-, CD11b+) were separated into three subsets: monocytes/macrophages (CD11c-, Ly6G-), neutrophils (CD11c-, Ly6G+), and dendritic cells (CD11c+). |

☒ Tick this box to confirm that a figure exemplifying the gating strategy is provided in the Supplementary Information.
