# Supplementary material for: TBK1-associated adapters TANK and AZI2 protect mice against TNF-induced cell death and severe autoinflammatory diseases
Source: Nat Commun. 2024 Nov 19;15:10013. doi: 10.1038/s41467-024-54399-4 (PMC11576971; doi:10.1038/s41467-024-54399-4)
Supplement: Supplementary file 4 — Description of Additional Supplementary Files [file 41467_2024_54399_MOESM4_ESM.pdf]

Description of Additional Supplementary Files

Supplementary Data 1

Description: Role of TANK and AZI2 in regulating TNF-induced gene expression.

Supplementary Data 2

Description: The list of reagents, animal strains, cell lines, and recombinant proteins used in this study.
